# Rapid Preparation of Spherical Granules via the Melt Centrifugal Atomization Technique

**DOI:** 10.3390/pharmaceutics11050198

**Published:** 2019-04-30

**Authors:** Yan Yang, Nan Zheng, Xiaoyue Wang, Ryan Ivone, Weiguang Shan, Jie Shen

**Affiliations:** 1 College of Pharmaceutical Science, Zhejiang University of Technology, Hangzhou 310014, China; yangyan10@zjut.edu.cn (Y.Y.); znde3344@163.com (N.Z.); ShiraureyYue@163.com (X.W.); 2 College of Pharmacy, University of Rhode Island, Kingston, RI 02881, USA; ryan_ivone@my.uri.edu

**Keywords:** melt centrifugal atomization (MCA), spherical granules, melt rheology, immediate release, moisture absorption

## Abstract

Granules with superior fluidity and low moisture absorption are ideal for tableting and capsule filling. Melt granulation as a solvent-free technology has attracted increasing interest for the granulation of moisture-sensitive drugs. The objective of the present study was to develop a solvent-less and high throughput melt granulation method via the melt centrifugal atomization (MCA) technique. The granule formability of various drugs and excipients via MCA and their dissolution properties were studied. It was found that the yield, fluidity, and moisture resistance of the granules were affected by the drug and excipient types, operation temperature, and collector diameter. The drugs were in an amorphous state in pure drug granules, or were highly dispersed in excipients as solid dispersions. The granules produced via MCA showed an improved drug dissolution. The present study demonstrated that the solvent-free, one-step, and high-throughput MCA approach can be used to produce spherical granules with superior fluidity and immediate drug release characteristics for poorly water-soluble and moisture-sensitive therapeutics.

## 1. Introduction 

Granules exhibiting superior fluidity are ideal for tableting and capsule filling. Compared with conventional granulation techniques, such as grinding and spray drying [1,2], melt granulation via a series of solvent-free techniques presents an alternative granulation approach suitable for moisture-sensitive drugs [3]. The melt granulation technique circumvents the need for a drying process, providing a more economical and environmentally sustainable process. Currently, spray congealing is the most commonly reported melt granulation method used to prepare drug-loaded granules. During the melting process, the state of the drug is often transformed from crystalline to amorphous. Consequently, an immediate drug release can be achieved using hydrophilic carriers such as mannitol and hydroxypropyl methylcellulose [4,5], whereas, sustained release can be obtained using hydrophobic carriers such as paraffin wax [6]. However, complex instrumentation and operation procedures have hindered the widespread use of this technique. 

An alternative approach for melt granulation is melt centrifugal atomization (MCA), in which a centrifugal force is employed to stretch the melt jet into molten droplets, which immediately solidify into spherical granules [7]. As a one-step and high-throughput approach to prepare uniform spherical granules, MCA has been used in the field of metal granule preparation [8]. However, the application of MCA is not directly transferrable to the pharmaceutical industry, because existing instruments are operated at temperatures that are much higher than the melting temperatures of active pharmaceutical ingredients and excipients. Whether MCA can be used for the one-step formation of drug-loaded solid dispersion granules with immediate drug release characteristics has not been previously reported. 

In our previous study, drug-loaded fibers were prepared via centrifugal met spinning, and the mechanism of fiber formation was investigated [9]. The present study aims to produce drug-loaded granules via the MCA technique using an in-house device. Various drugs and pharmaceutical excipients were tested for granule formability, and melt rheology was employed in order to understand the granulation process. The effects of drug and excipient type, operation temperature, and collector diameter on the morphology and properties (e.g., dissolution and moisture absorption) of the produced granules were investigated. 

## 2. Materials and Methods

### 2.1. Materials

Indomethacin (IND) was supplied by XiYinHe Chemical Co., Ltd. (Wuhan, China). Nifedipine (NF), tinidazole (TNZ), and metoprolol tartrate (MT) were purchased from YuanCheng Pharmaceutical Co., Ltd. (Wuhan, China). Astragalus polysaccharide (AP; 90% extracts) was purchased from Xian Jinheng Chemical Co., Ltd. (Xi’an, China). Polyethylene glycol (PEG; 6000 Da) was purchased from XiLong Chemical Co., Ltd. (Shantou, China). Mannitol was provided by Merck KGaA (Darmstadt, Germany). Sucrose was provided by Guangdong Guanghua Sci-Tech Co., Ltd. (Shantou, China). Soluplus^®^ (SOL) was provided by BASF (Ludwigshafen, Germany). Stearic acid (SA) was purchased from Wenzhou Chemical Material Factory (WenZhou, China). Glycerol monostearate/distearate (GMDS) was purchased from Er-Kang Biological Technology Co., Ltd (Changsha, China). Paraffin was purchased from Shanghai Hualing Kuangfu Co., Ltd. (Shanghai, China). Eudragit^®^ RL PO (RL) was supplied by Evonik Rohm Co., Ltd. (Darmstadt, Germany).

### 2.2. Preparation of Spherical Granules

**Figure 1:**
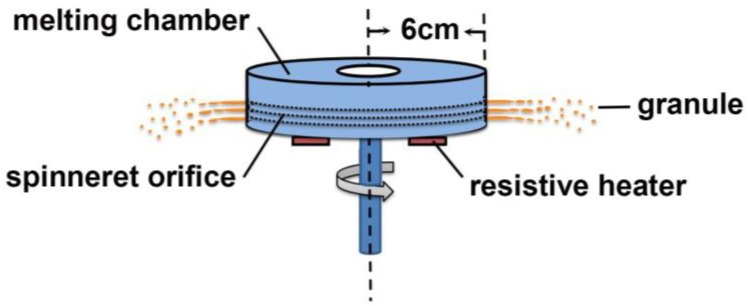
Illustration of the granulation process via melt centrifugal atomization (MCA).

Spherical granules were prepared using an in-house MCA device, as illustrated in Figure 1. The size of the orifices on the side wall of the device was about 50 μm. Pure drug granules (i.e., IND, NF, TNZ, MT, and AP), pure excipient granules (i.e., PEG, mannitol, sucrose, SOL, SA, GMDS, paraffin, and RL), and granules containing both drug and excipient were produced. The materials were mixed in a mortar for 10 min in varying ratios, and were added into the melting chamber. The melting chamber was heated at a rate of 5 °C/min until the materials inside them started to melt, and then it was immediately rotated at a speed of 3000 rpm. The molten materials with different amounts (i.e., ~2 or 5 g) were centrifuged through the spinneret orifices on the side wall, and the products were collected at different collection distances (i.e., 5 and 19 cm) using concentric barrels with different radii (i.e., 11 and 25 cm).

### 2.3. Characterization of Granules

The morphology of the products collected was observed using a stereomicroscope (ZY-HD1400, ZongyanWeiye, Shenzhen, China) in the magnification of 200×. The yield of the granules was determined using a gravimetric sieving method. The granules passed through the 65-mesh screen were weighed and the yield was calculated. About 5 g of the granule samples were dispensed through a stainless-steel funnel onto a circular base plate to form a conical heap. The angle of repose was calculated by determining the radius and height of the cone [10]. 

The moisture absorption study was conducted according to the method described in EP 9.0. Glass weighing bottles were equilibrated in 25 ± 1 °C, with a saturated ammonium sulfate solution (RH 80% ± 2%) for 24 h, and were accurately weighed. The particle samples were transferred into the bottles to form a 1-mm thick layer, and the bottles were kept under the same temperature and humidity conditions as described above. At pre-determined time intervals, the weight and mass percent increase were accurately determined.

### 2.4. Thermal Analysis

Thermal gravimetric analysis (TGA) was conducted to determine the thermal decomposition temperature (*T_d_*). Accurately weighed samples (~20 mg) (i.e., IND, NF, TNZ, and MT) were heated from 25 to 400 °C at a constant rate of 10 °C/min under a nitrogen purge of 20 mL/min using a TG analyzer (Q5000, TA instrument, New Castle, DE, USA) [11]. The TGA curves were drawn subsequently.

Differential scanning calorimetry (DSC) was conducted to determine the melting temperature (*T_m_*). Accurately weighed samples (~5 mg) were analyzed using a differential scanning calorimeter (DSC1, Mettler-Toledo, Greifensee, Switzerland) [12] at a temperature range of 25–150 °C for TNZ and MT, and 25–200 °C for NF and IND, respectively. The heating rate was 10 °C/min under a nitrogen purge of 50 mL/min. 

### 2.5. Melt Rheological Study

The melt rheological properties of the representative drugs and excipients were determined via an Anton Paar Physica Rheometer (MCR 302, Anton Paar, Graz, Austria). NF and TNZ were selected as representative model drugs, as NF has the highest melting point and TNZ has the lowest melting point out of all of the drugs studied. In addition, PEG with excellent granule formability, and GMDS and SOL that form fibers rather than granules, were selected as representative excipients. There were 25.0 mm parallel plates used in an oscillation mode, with a gap distance of 0.3 mm [13]. Frequency sweep tests were performed at an angular frequency of 10 rad/s with a strain amplitude of 0.5%. Complex viscosity (η*) measurements were taken every 5 s. When the samples reached their equilibrium temperature (NF = 190 °C, TNZ = 135 °C, PEG = 100 °C, GMDS = 80 °C, and SOL = 200 °C), the test was commenced at a cooling rate of 0.8 °C/min. 

### 2.6. X-ray Diffraction (XRD) Analysis

XRD analysis of the samples (i.e., raw IND, IND granules, raw NF, and NF granules) was performed in an X-ray diffractometer (D/max 2550/PC, Rigaku, Tokyo, Japan) with Cu Kα_1_ radiation. The samples were scanned at 40 kV and 40 mA, in the 2θ range of 5–50° at 10°/min [14].

### 2.7. In Vitro Dissolution Study

The in vitro dissolution testing was conducted using a USP apparatus II method at 37 ± 0.5 °C, with the paddle speed set at 100 rpm. Then, 500 mL of release media (i.e., phosphate buffer solution (pH 7.2)) for IND and 0.5% (*w*/*v*) SLS solution for NF, were used. At predetermined time intervals, release samples were withdrawn, filtered, and analyzed using a spectrophotometer (UV-2450, Shimadzu, Japan). The calibration curve for IND at 320 nm was *A* = 0.0193*C* (*R* = 1.0000), and the calibration curve for NF at 333 nm was *A* = 0.0149*C* − 0.0048 (*R* = 0.9999). Both methods were validated for accuracy, precision, and recovery (data not shown). The cumulative drug release at different time points was calculated, and the in vitro dissolution curves were drawn. 

## 3. Results and Discussion 

### 3.1. Influence of Drugs and Excipients on Granule Formability

**Figure 2:**
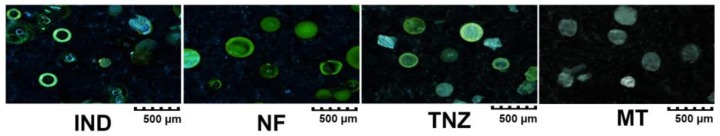
Morphology of indomethacin (IND), nifedipine (NF), tinidazole (TNZ), and metoprolol tartrate (MT) granules observed using a stereomicroscope.

Poorly water-soluble drugs (i.e., IND and NF), water-soluble drugs (i.e., TNZ and MT), and hygroscopic natural product extracts (i.e., AP) were studied in order to understand the effect of the drugs on the granule formability via MCA technique. As shown in Table 1, the *T_m_* determined via DSC was found to be MT < TNZ < IND < NF, and the *T_d_* determined via TGA was found to be MT < NF < TNZ = IND. The operation temperature of the MCA process should be above *T_m_* and below *T_d_*. The natural product extract AP had no obvious melting temperature and carbonized easily. Therefore, pure AP granules could not be produced via the MCA process. As shown in Figure 2, the collected pure drug granules exhibited a spherical morphology with a particle size of 100–200 μm. Among them, the IND granules were transparent and the other three drug-loaded granules were opaque, presenting the color of the drugs themselves. In addition, the obtained pure drug granules had a low angle of repose (IND = 29.7°, NF = 29.0°, TNZ = 30.0°, and MT = 30.7°) suggesting superior fluidity. 

**Figure 3:**
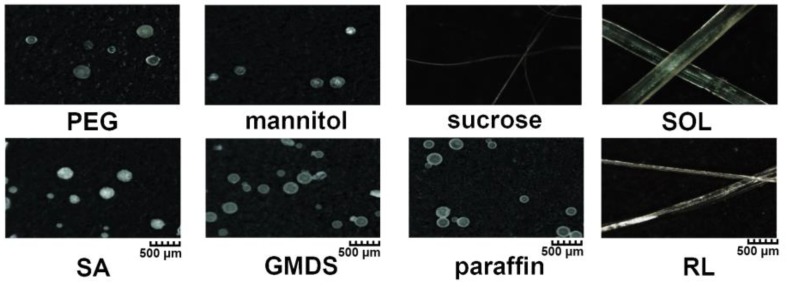
Morphology of the products composed of polyethylene glycol (PEG), mannitol, sucrose, Soluplus^®^ (SOL), stearic acid (SA), glycerol monostearate/distearate (GMDS), paraffin, and Eudragit^®^ RL PO (RL) prepared via MCA.

Commonly used hydrophilic excipients (such as PEG, mannitol, sucrose, and SOL) and hydrophobic excipients (such as SA, GMDS, paraffin, and RL) were studied to understand the effect of different excipients on granule formability via MCA. *T_m_* and *T_d_* of the excipients were obtained from the literature [4,6,15,16,17,18,19,20,21,22] and are listed in Table 1. Compared with PEG, paraffin, SA, and GMDS with low *T_m_* (56–61 °C), mannitol and sucrose had much higher *T_m_* (166–185 °C). SOL and RL had no obvious *T_m_*, and their softening temperature observed during the MCA process was about 150 and 160 °C, respectively. As shown in Figure 3, the products made of PEG, mannitol, SA, GMDS, and paraffin were typically spherical granules, while the products made of sucrose, SOL, and RL were fibers with different diameters. Moreover, the obtained granules composed excipients also had a low angle of repose (PEG = 29.3°, mannitol = 29.4°, SA = 31.0°, GMDS = 29.5°, and paraffin = 29.7°), suggesting superior fluidity.

**Figure 4:**
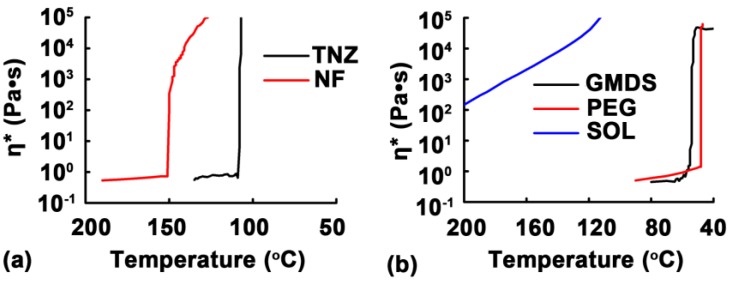
Melt complex viscosity (η*) curves of (**a**) nifedipine (NF) and tinidazole (TNZ); and (**b**) polyethylene glycol (PEG), Soluplus^®^ (SOL), and glycerol monostearate/distearate (GMDS).

A melt rheology study of the drugs and excipients was conducted in order to elucidate the formation mechanism of the granules during the MCA process. As shown in Figure 4a, TNZ and NF had low η* values (<1 Pa·s) at their melting temperatures. Upon leaving the spinneret orifices, the melts with a low viscosity easily broke into melt droplets and rapidly solidified into spherical granules. A similar phenomenon was previously reported [23], in which a solution jet broke into spherical droplets during centrifugation. As shown in Figure 4b, the melt viscosity curves of PEG and GMDS were similar to those of TNZ and NF, leading to the formation of spherical granules (Figure 3). On the other hand, the η* value of SOL gradually increased from 147 to 42,990 Pa·s when cooled from 200 to 120 °C. As a result, the molten SOL had a high η* value upon leaving the spinneret orifices, and was stretched into fibers instead of spherical granules. This was consistent with the results reported in the literature [24], where an increase in the liquid viscosity resulted in the formation of ligaments instead of droplets during the granulation process. This suggested that maintaining a low η* within a wide temperature range facilitated in the formation of granules via MCA. Crystalline or waxy excipients with specific melting temperatures (e.g., PEG or SA) seemed to be suitable for producing granules via MCA. Whereas, amorphous polymers (e.g., SOL and RL) may be more suitable for preparing fibers via either centrifugal met spinning [9] or MCA.

### 3.2. Influence of Operating Parameters on the Morphology and Yield of Granules

**Figure 5:**
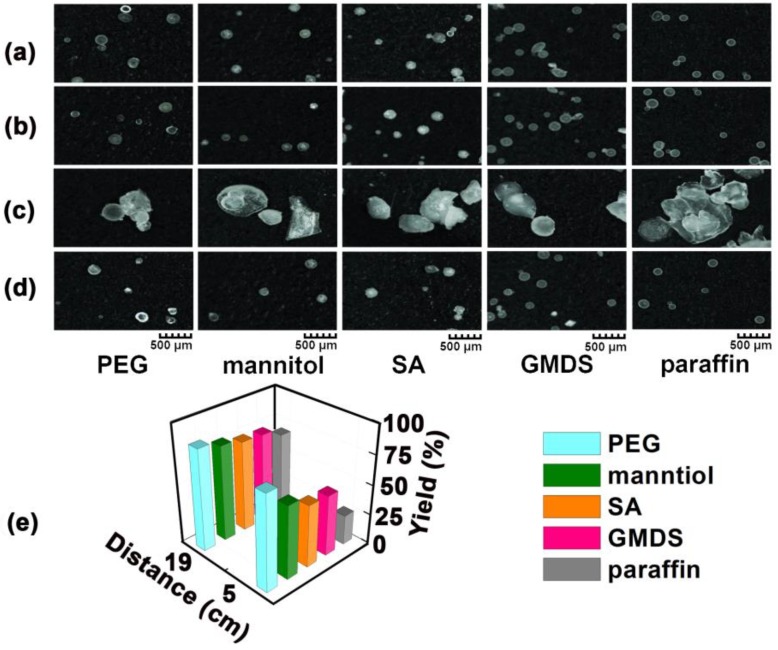
Morphology of products collected with 2 g of materials at a distance of (**a**) 5 cm and (**b**) 19 cm; and with 5 g of materials at a distance of (**c**) 5 cm and (**d**) 19 cm; (**e**) The yield of the granules with a constant material feeding amount of 5 g. PEG—polyethylene glycol; SA—stearic acid; GMDS—glycerol monostearate/distearate.

The effect of collecting the distance on the morphology of the granules is shown in Figure 5a–d. When a small amount of material (about 2 g) was used, the products collected at a short distance of 5 cm were similar to those collected at a long distance of 19 cm, existing mainly as spherical granules (Figure 5a,b). During the MCA process, the particle morphology is mainly governed by a centrifugal force, viscous force, and surface tension exerted on the molten materials [25]. When keeping the melting temperature and rotating speed constant, the particle size of the products was mainly affected by the material properties e.g., viscosity, density, and surface tension. However, when a larger amount of material (about 5 g) was used, the products collected at a short distance (5 cm) were mainly an irregular-shaped large mass (Figure 5c). Granules easily agglomerated as a result of the incomplete solidification at a short collecting distance. On the other hand, the products collected at a long distance (19 cm) remained spherical (Figure 5d). With a constant material feeding amount of 5 g, the granule yield was increased when the collecting distance was increased from 5 to 19 cm (Figure 5e). A longer collecting distance resulted in a more complete solidification, and hence the formation of spherical granules and higher yield.

**Figure 6:**
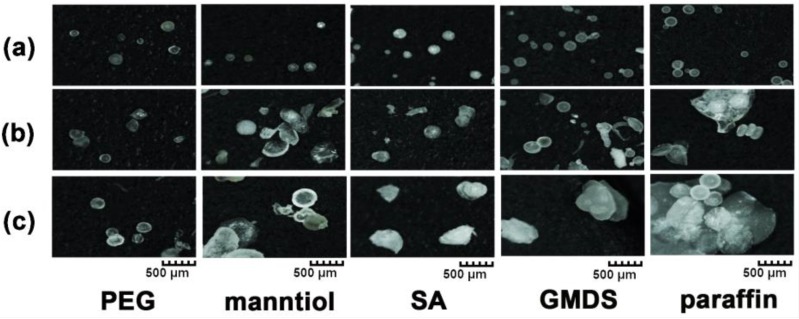
Influence of different operation temperatures including (**a**) *T_m_*; (**b**) 15°C above *T_m_* and (**c**) 30 °C above *T_m_* on the morphology of products. PEG—polyethylene glycol; SA—stearic acid; GMDS—glycerol monostearate/distearate.

The effect of the operation temperature on the morphology and yield of the granules is shown in Figure 6. In general, it takes a longer time for most excipients (e.g., mannitol, SA, GMDS, and paraffin) to cool down and to solidify when heated to a higher temperature. When the operation temperature was 15 or 30 °C above *T_m_*, incomplete particle solidification and agglomerate formation in the collector were observed, resulting in a reduced granule yield. Therefore, the optimized MCA process conditions were as follows: 5 g of material centrifugated at an operation temperature of *T_m_*, and a collecting distance of 19 cm.

### 3.3. Moisture Absorption of Granules

**Figure 7:**
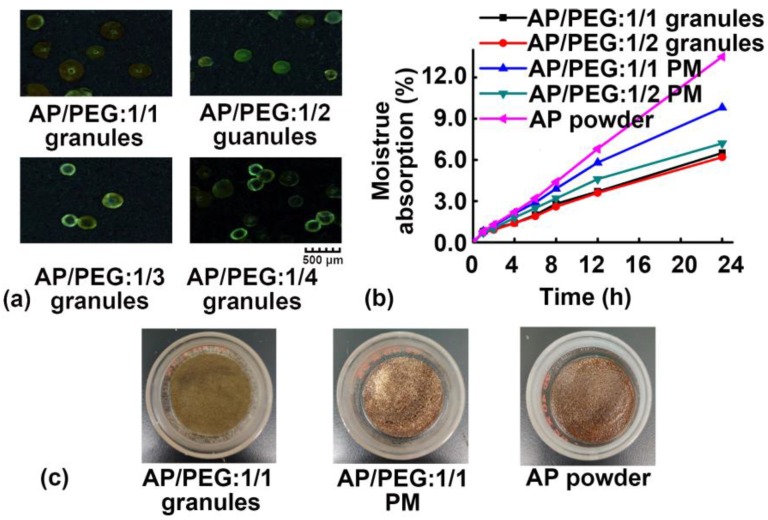
(**a**) Typical morphology of astragalus polysaccharide (AP)/polyethylene glycol (PEG) granules with different ratios at 0 h observed by a stereomicroscope. (**b**) Moisture absorption curves of granules produced via melt centrifugal atomization (MCA), physical mixture (PM) of AP and PEG, and AP during 24 h; and (**c**) their typical appearance at 24 h.

As discussed in Section 3.1 above, the natural product extract AP itself had no granule formability via MCA. As an excipient with a high granule formability, PEG was chosen as a carrier, and mixed with a spray-dried AP powder to prepare AP/PEG granules via MCA. As shown in Figure 7a, the drug/excipient ratio had little effect on the morphology of the AP/PEG granules. As shown in Figure 7b, the moisture absorption of the AP/PEG granules prepared via MCA was significantly decreased compared with those of the spray-dried AP powder and the corresponding physical mixture (PM). The moisture absorption at 24 h of the AP/PEG (1/1, *w*/*w*) granules, PM (AP/PEG:1/1, *w*/*w*), and AP powder was 6.49%, 9.80%, and 13.51%, respectively. The AP/PEG granules exhibited a larger particle size and hence a reduced specific surface area compared with the AP powder, resulting in less water absorption. Further increasing the PEG concentration (AP/PEG:1/2, *w*/*w*) had no obvious effect on the moisture absorption, suggesting that the hygroscopic property of AP may be size dependent. As shown in Figure 7c, the appearance of the AP/PEG (1/1, *w*/*w*) granules did not change after being exposed to a high humidity (RH 80% ± 2%) for 24 h, while the wetting phenomenon was found in the pure AP powder and PM (AP/PEG:1/1, *w*/*w*) at 24 h. The AP/PEG granules prepared via MCA can be further processed into tablets and capsules, and are expected to have better stability during preparation and storage compared with the AP powder. 

### 3.4. Immediate Release Characteristics of Granules

**Figure 8:**
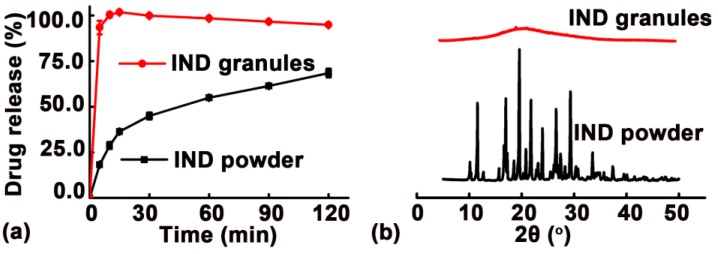
(**a**) In vitro dissolution profiles and (**b**) XRD results of indomethacin (IND) granules prepared via melt centrifugal atomization (MCA) and IND powder.

As shown in Figure 8a, the dissolution of IND granules prepared via MCA was faster than that of the IND powder. The cumulative IND released from the granules at 5 min was over 90%. Further XRD results (Figure 8b) demonstrated that the IND transitioned from a crystalline to amorphous state in the IND granules during the MCA process, resulting in an immediate drug dissolution characteristic.

**Figure 9:**
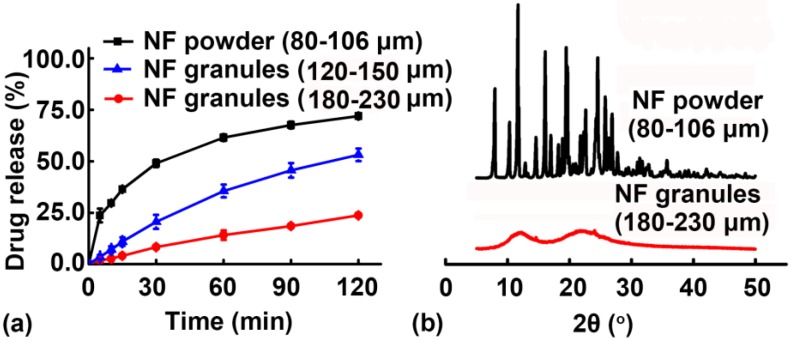
(**a**) In vitro dissolution profiles and (**b**) XRD results of nifedipine (NF) powder and NF granules prepared via melt centrifugal atomization (MCA).

It was unexpected that no immediate drug release characteristic was found in the NF granules prepared via MCA. The influence of particle size on the NF dissolution is shown in Figure 9a. The results revealed that NF released from small NF granules (120–150 μm) prepared via MCA was faster than that from large NF granules (180–230 μm) prepared via MCA, but slower than that from the drug powder (80–106 μm). Although, the XRD results (Figure 9b) demonstrated that the crystallinity of NF was reduced in the NF granules compared to that in the NF powder. It appeared that the main factor affecting NF dissolution was particle size.

**Figure 10:**
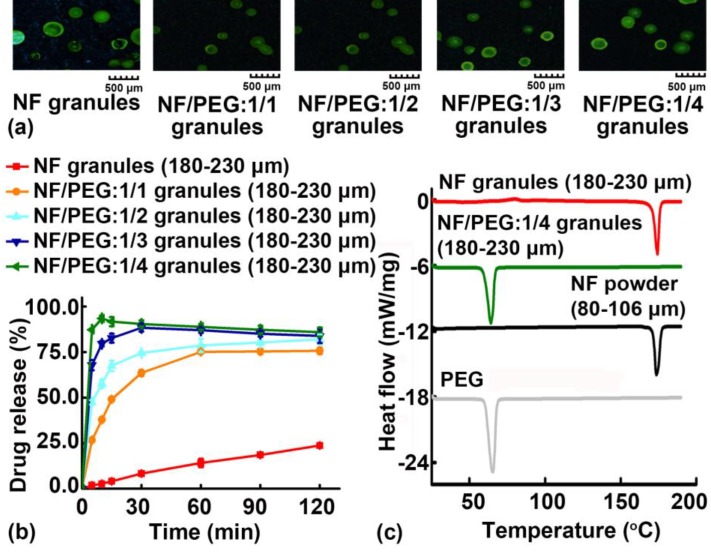
(**a**) Typical morphology observed by stereomicroscope and (**b**) in vitro dissolution profiles of nifedipine (NF)/polyethylene glycol (PEG) granules with different ratios. (**c**) DSC curves of NF granules, NF/PEG: 1/4 granules, NF powder, and PEG.

In order to improve the dissolution of NF granules, PEG was added as a hydrophilic carrier. It was observed that the drug/excipient ratio had no obvious effect on the morphology and shape of the NF/PEG granules (Figure 10a). However, the drug/excipient ratio had a significant influence on the NF dissolution behavior (Figure 10b). Granules containing a higher PEG content resulted in an enhanced dissolution rate and extent of NF. The drug dissolution of the NF/PEG granules at 10 min was up to 79.62% and 93.51% for the granules with NF/PEG ratios of 1/3 and 1/4, respectively. DSC studies (Figure 10c) confirmed that the resulting system was an amorphous or molecular solid dispersion, and thereby, immediate NF release was attained. An effort was also made to produce granules with sustained release characteristics, using TNZ as the model drug and GMDS as the hydrophobic carrier. Even when the GMDS was as high as 80%, no sustained TNZ release was obtained (data not shown). These results suggested that the MCA technique can be an effective way to produce spherical granules with enhanced dissolution profiles for poorly water-soluble therapeutics such as NF.

## 4. Conclusions

A solvent-less, one-step, and high throughput MCA method was developed to prepare spherical granules. The produced granules had a high drug loading, superior fluidity, low moisture absorption, and immediate release properties. Maintaining a low molten viscosity in a wide temperature range was found to be critical for the formation of spherical granules. The results demonstrated that the one-step MCA approach could be used to prepare spherical granules with immediate release characteristics for poorly water-soluble and moisture-sensitive drugs.

## Figures and Tables

**Table 1:** Melting temperature (*T_m_*) and thermal decomposition temperature (*T_d_*) of drugs (i.e., indomethacin (IND), nifedipine (NF), tinidazole (TNZ), and metoprolol tartrate (MT)), hydrophilic excipients (i.e., polyethylene glycol (PEG), mannitol, sucrose, and Soluplus^®^ (SOL)) and hydrophobic excipients (i.e., stearic acid (SA), glycerol monostearate/distearate (GMDS), paraffin, and Eudragit^®^ RL PO (RL)).

Drugs	*T_m_* (°C)	*T_d_* (°C)	Hydrophilic Excipients	*T_m_* (°C)	*T_d_* (°C)	Hydrophobic Excipients	*T_m_* (°C)	*T_d_* (°C)
IND	162	220	PEG [15]	60	290	SA [16]	60	250
NF	174	215	mannitol [4]	166	270	GMDS [17,18]	56	125
TNZ	124	220	Sucrose [19]	185	210	paraffin [6,20]	61	235
MT	123	180	SOL [21]	/	250	RL [22]	/	170

## Nomenclature

MCA	melt centrifugal atomization
IND	indomethacin
NF	nifedipine
TNZ	tinidazole
MT	metoprolol tartrate
AP	astragalus polysaccharide
PEG	polyethylene glycol
SOL	Soluplus^®^
SA	stearic acid
GMDS	glycerol monostearate/distearate
RL	Eudragit^®^ RL PO
TGA	thermal gravimetric analysis
*T_d_*	thermal decomposition temperature
DSC	differential scanning calorimetry
*T_m_*	melting temperature
*η**	complex viscosity
XRD	X-ray diffraction
PM	physical mixture
